# Altered Frequency and Phenotype of HLA-G-Expressing DC-10 in Type 1 Diabetes Patients at Onset and in Subjects at Risk to Develop the Disease

**DOI:** 10.3389/fimmu.2021.750162

**Published:** 2021-10-01

**Authors:** Giada Amodio, Alessandra Mandelli, Rosalia Curto, Paola M. V. Rancoita, Angela Stabilini, Riccardo Bonfanti, Maurizio de Pellegrin, Emanuele Bosi, Clelia Di Serio, Manuela Battaglia, Silvia Gregori

**Affiliations:** ^1^ Mechanisms of Peripheral Tolerance Unit, San Raffaele Telethon Institute for Gene Therapy (SR-Tiget), Istituto di Ricovero e Cura a Carattere Scientifico (IRCCS) San Raffaele Scientific Institute, Milan, Italy; ^2^ Immune-Mediated Diseases Unit: From Pathogenesis to Treatment, Diabetes Research Institute (DRI), Division of Immunology, Transplantation, and Infectious Diseases, IRCCS San Raffaele Scientific Institute, Milan, Italy; ^3^ University Center for Statistics in the Biomedical Sciences (CUSSB), Vita-Salute San Raffaele University, Milan, Italy; ^4^ Department of Pediatrics and Neonatology, IRCCS San Raffaele Hospital, Milan, Italy; ^5^ Department of Pediatric Orthopedics, IRCCS San Raffaele Hospital, Milan, Italy; ^6^ Department of Internal Medicine, IRCCS San Raffaele Hospital, Milan, Italy; ^7^ TrialNet Clinical Center, IRCCS San Raffaele Hospital, Milan, Italy

**Keywords:** DC-10, type 1 diabetes (T1D), HLA-G, tolerogenic dendritic cells, pro-inflammatory myeloid cells, first-degree relatives (FDRs)

## Abstract

Type 1 diabetes (T1D) is a chronic autoimmune disease resulting in progressive destruction of β-cells. Several factors affecting lymphocyte and antigen-presenting cells, including dendritic cells (DCs), contribute to defective maintenance of tolerance in T1D. DC-10 are a subset of human DCs involved in IL-10-mediated tolerance. A precise monitoring of DC-10 in the peripheral blood is possible thanks to the discovery of specific biomarkers. DC-10, being cells that naturally express HLA-G, may be used for the appropriate staging of the disease. By enumerating and phenotypically characterizing DC-10 in the peripheral blood of subjects at different stages of T1D development—first-degree relatives (FDRs) of T1D patients, without (Ab^neg^) or with (Ab^pos^) autoantibodies, T1D patients at onset, and age-matched healthy controls (HCs)—we showed that DC-10 contain a high proportion of HLA-G-expressing cells as compared with monocytes. We reported that a low frequency of DC-10 during disease development is paralleled with the increased proportion of pro-inflammatory cDC2 cells. Moreover, DC-10 number and phenotype differ from Ab^neg^ FDRs, Ab^pos^ FDRs, and T1D patients compared with HCs, and DC-10 from T1D patients express low levels of CD83. Finally, multiple regression analysis, considering DC-10 and HLA-G-related parameters, showed that Ab^neg^ FDRs are more similar to subjects with autoimmunity than to HCs. This is the first demonstration that impairment in DC-10 number and phenotype, specifically CD83 expression, is associated with risk of developing T1D, suggesting a possible use of CD83^+^ DC-10 to stratify individuals at risk of T1D in conjunction with classical prognostic factors.

## Introduction

Type 1 diabetes (T1D) is a T cell-mediated autoimmune disease characterized by progressive destruction of pancreatic β cells (1). Both genetic and environmental factors, by affecting lymphocyte activities (2) and antigen-presenting cells (APCs), contribute to defective induction/maintenance of tolerance in T1D. Dendritic cells (DCs) are professional APCs implicated in dictating the activation and differentiation of T cells. Conventional (c)DCs promote the initiation of pathogenic T-cell responses, whereas non-activated DCs, or specialized subsets of DCs, termed tolerogenic (tol)DCs, induce or activate regulatory T cells leading to tolerance (3). Aberrant activation of cDCs or defects in tolDC activities contribute to breaking self-tolerance in T1D (4). Activated cDCs, which stimulate auto-reactive T cells and secrete pro-inflammatory cytokines, and defective tolDCs have been reported in pancreatic islets and lymph nodes of T1D patients (5, 6). Moreover, cDC subsets have been described in the peripheral blood of T1D patients (4). The lack of specific biomarkers for identifying tolDCs has limited their identification and study in T1D patients.

DC-10 are a subset of HLA-G-expressing tolDCs, which induce adaptive T regulatory type 1 (Tr1) cells *in vitro via* the IL-10-dependent HLA-G/ILT4 pathway (7). HLA-G is a well-recognized immune-modulatory molecule that regulates innate and adaptive immune responses and promotes tolerance (8). Polymorphisms located at the 3′ un-translated region (UTR) of *HLA-G* locus, which can arrange in a limited number of haplotypes and finely tune the HLA-G expression (9), have been associated with T1D (10–12). Moreover, the tolerogenic potential of DC-10 is associated with genetic variations of the *HLA-G* locus encoding for elevated levels of protein expression (13). The discovery that DC-10 are present *in vivo* (7, 14), modulate T-cell responses, induce Tr1 cells *in vitro* in a HLA-G-dependent manner, and are associated with tolerance (15) prompted us to postulate that DC-10 represent an important subset of naturally occurring HLA-G-expressing DCs involved in promoting tolerance in T1D than can be used as biomarker for staging the disease in conjunction with classical prognostic factors. The discovery of CD141, CD163, CD14, and CD16 as specific DC-10 biomarkers (14) allows a precise monitoring of DC-10 in the peripheral blood and tissue.

In this study, we evaluated the presence and frequency of HLA-G-expressing DC-10 in the peripheral blood of first-degree relatives (FDRs) of T1D patients with negative (Ab^neg^) or positive (Ab^pos^) serology, T1D patients at onset, and age-matched healthy controls (HCs). We show that DC-10 number and phenotype differ from Ab^neg^ FDRs, Ab^pos^ FDRs, and T1D patients, compared with HCs. In addition, in T1D patients, the ratio between tolerogenic DC-10 and pro-inflammatory cDC2 is weighted toward the immunogenic compartment. Finally, comparison of six variables associated with DC-10 and HLA-G revealed that Ab^neg^ FDRs are different from HCs and that a progressive decrease of CD83^+^ DC-10 percentage is associated with disease development.

## Materials and Methods

### Study Subjects

Adult HCs (>18 years) were admitted to the “Servizio Medicina Preventiva” at the San Raffaele University Hospital, Milan, Italy. Pediatric HCs (≤18 years) were children with no immune system-related diseases undergoing surgery only for congenital disease at the Orthopedic Pediatric Department at the San Raffaele Hospital. Both adult and pediatric HCs had no family members with T1D.

Adult T1D patients were recruited at the Endocrinology Department. For genetic analysis, both new-onset (blood withdrawal within 3 months from diagnosis) and long-standing adult T1D individuals were included in the study. Pediatric T1D subjects were enrolled at the Pediatric and Neonatology Department at the San Raffaele Hospital. Only children with a blood withdrawal within the first 10 days after diagnosis (new-onset T1D) were included in the study. The diagnosis of T1D was made based on sustained hyperglycemia (documented by repeated glucose measurements and HbA1c measurement), fasting C-peptide levels, 1.0 ng/ml, or the presence of at least one islet-specific autoantibody (16, 17).

FDRs of T1D patients were recruited under the umbrella of the Type 1 diabetes TrialNet Pathway to Prevention Trial (TN01) at the TrialNet Clinical Center of the San Raffaele Hospital. These subjects were divided in two groups based on the presence of circulating islet-specific autoantibodies (e.g., GAD65, IA2, mIAA, or ZnT8) at blood withdrawal: FDRs without (Ab^neg^) or with at least one (Ab^pos^) autoantibody.

Human peripheral blood was collected upon informed consent in accordance with the Declaration of Helsinki and with local ethical committee approvals: IRB#DRI-003 for HCs and T1D patients and IRB#NHPROT32803-TN01 for Ab^neg^ and Ab^pos^ subjects. For pediatric subjects, the informed consent has been provided by parents. All the individuals included in the study were European Caucasians, and samples were collected from 2016 to 2017. A full blood count laboratory test was performed for all the samples received. Subjects with ongoing infections were excluded from the analysis.

Characteristics of subjects enrolled in the study are listed in (**Table 1**) and in (**Table 2**).

**Table 1:** Characteristics of subjects enrolled in the study.

Variables	HCs(n = 40)	Ab^neg^ FDRs(n = 37)	Ab^pos^ FDRs(n = 21)	T1D (n = 22)
Age (years)	12 (5–17)	11 (4–17)	10 (5–17)	12 (3–17)
Female sex, n (%)	18 (45)	18 (49)	9 (43)	8 (36)
# of auto Abs	N.A.	0	3 (1–5)	3 (0–4) [4 missing]
Days from diagnosis	N.A.	N.A.	N.A.	5 (2–10)
HbA1c (%)	N.A.	N.A.	5.3 (4.4–5.8) [2 missing]	11.9 (9.4–15.4)
C-peptide (ng/ml)	N.A.	N.A.	N.A.	0.32 (0.02–1.27) [1 missing]

The sample includes individuals ≤17 years old. Continuous variables are presented as median (min–max); categorical variables are presented as frequency (%). Clinical parameters that were not available for one or more Ab^pos^ FDRs and T1D patients are indicated as missing.

N.A., not available; HCs, healthy controls; FDRs, first-degree relatives; T1D, type 1 diabetes.

**Table 2:** Characteristics of subjects enrolled for genetic studies.

Variables	HCs(n = 78)	Ab^neg^ FDRs(n = 97)	Ab^pos^ FDRs(n = 52)	T1D (n = 43)
Age (years)	14 (5–43)	13 (2–44)	15 (4–49)	13 (3–41)
Female sex, n (%)	33 (42)	53 (55)	25 (48)	20 (47)
# of auto Abs	N.A.	0	2 (1–5)	3 (0–4) [5 missing]
Days from diagnosis	N.A.	N.A.	N.A.	6 (0–6668)
HbA1c (%)	N.A.	N.A.	5.1 (4.4–6) [4 missing]	10.5 (5.4–15.4) [1 missing]
C-peptide (ng/ml)	N.A.	N.A.	N.A.	0.37 (0.00–1.30) [4 missing]

Continuous variables are presented as median (min–max); categorical variables are presented as frequency (%). Clinical parameters that were not available for one or more Ab^pos^ FDRs and T1D patients are indicated as missing.

N.A., not available; HCs, healthy controls; FDRs, first-degree relatives; T1D, type 1 diabetes.

### Flow Cytometry Analysis

The frequencies of DC-10 and other monocyte/myeloid populations, and the HLA-G, ILT4, CD83, and HLA-DR expression levels were assessed on EDTA peripheral blood within 6 h after blood withdrawal. In brief, 50 µl of antibody (Ab) mix was directly added to 180 µl of whole blood and incubated for 15 min at room temperature in the dark. The samples were then incubated for additional 13 min at room temperature in the dark with lysis buffer (EDTA 1 mM, KHCO_3_ 10 mM, and NH_4_Cl 150 mM) to perform red blood cell lysis, washed twice, and re-suspended in phosphate-buffered saline (PBS) (Sigma, CA, USA) with 2% fetal bovine serum (FBS) (Lonza, Italy). Cells were identified using a multiparametric approach based on the combination of monoclonal Abs (**Supplementary Table S1**). Samples were acquired within 12 h after the staining using a FACSCanto II flow cytometer (Becton Dickinson, Mountain View, CA). Samples were processed and stained in batches to reduce technical variability. Moreover, to ensure a standardized sample analysis and a reliable mean fluorescence intensity (MFI) evaluation among samples, flow cytometer was calibrated daily using 8-peak rainbow calibration particles (Spherotech, IL, USA) before sample acquisition. MFI was evaluated on the whole gated populations. Data were analyzed with FCS express v6 (*De Novo* Software, Glendale, CA), and quadrant markers were set accordingly to the relative fluorescence minus one (FMO) staining performed for all the donors analyzed.

### Statistical Analysis

**Figure 1:**
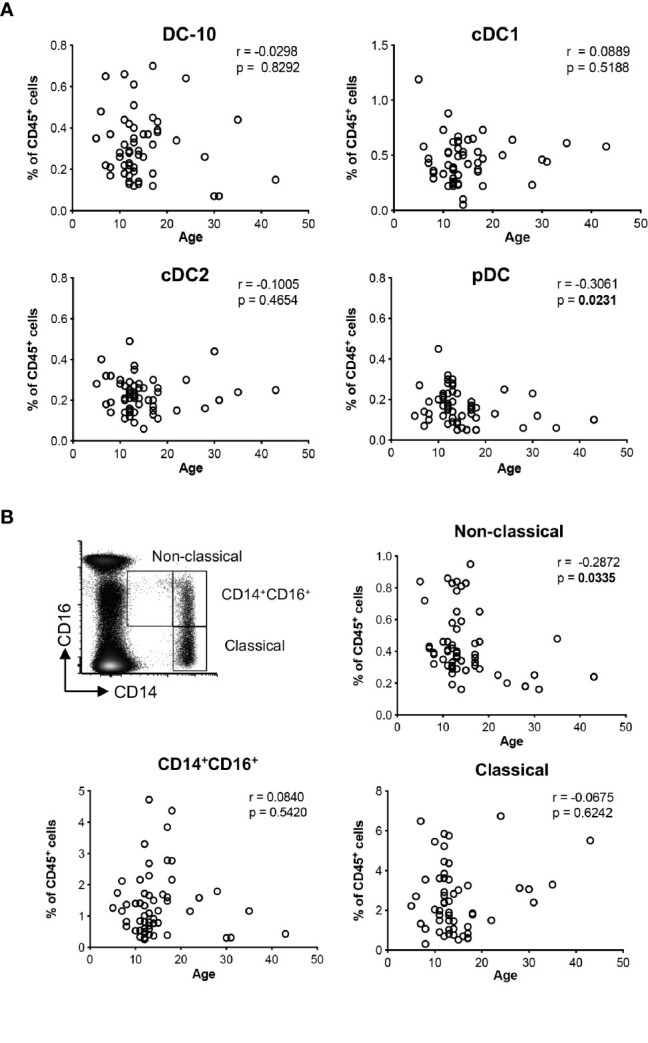
Distributions of the major dendritic cell and monocyte subsets with age. The percentage of DC-10 (CD11c^+^CD14^+^CD16^+^CD141^+^CD163^+^), cDC1 (CD11c^+^CD14^−^CD141^+^), cDC2 (CD11c^+^CD1c^+^), and pDC (CD11c^−^CD303^+^), and CD14^+^CD16^+^, and non-classical (CD14^low^CD16^+^, and classical (CD14^high^CD16^−^) monocytes were evaluated by multiparametric flow cytometry in the peripheral blood of healthy donors (HCs) at different ages. Scatterplot and Spearman’s correlation analyses between age and the indicated DC populations **(A)** and monocyte subsets **(B)** are shown. Each data point represents a donor (n = 55). Data distribution, Spearman’s rank correlation coefficient (r), and p-values are shown for each analysis. Statistically significant p-values are indicated in bold.

**Figure 2:**
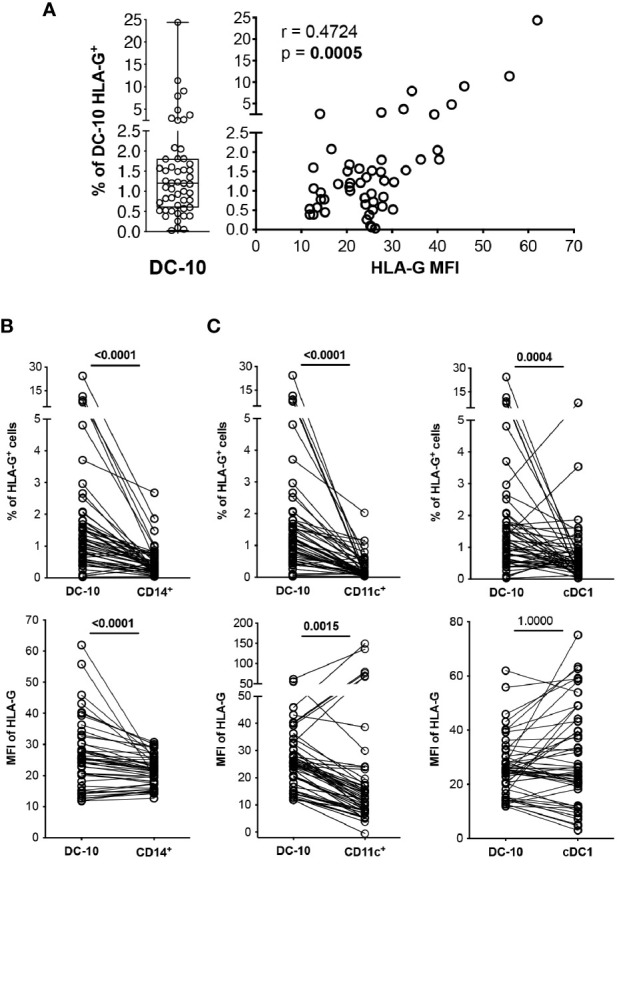
DC-10 contain a high proportion of HLA-G-expressing cells. DC-10 (CD11c^+^CD14^+^CD16^+^CD141^+^CD163^+^), CD14^+^, CD11c^+^, and cDC1 (CD11c^+^CD14^−^CD141^+^) cells were identified, and expression levels of HLA-G were evaluated by multiparametric flow cytometry in the peripheral blood of 51 healthy donors at different age. **(A)** Percentages of HLA-G-expressing DC-10. Each dot represents a single donor, line indicates median, and whiskers are minimum and maximum levels (left panel). Scatterplot and Spearman’s correlation analyses between the percentage of DC-10 expressing HLA-G and HLA-G mean fluorescence intensity (MFI) are shown. MFI was evaluated on the whole gated populations. Each data point represents a donor. Data distribution, Spearman’s rank correlation coefficient (r), and p-value are shown (right panel). **(B)** Percentages of HLA-G-expressing cells (top panel) and HLA-G MFI (bottom panel) on DC-10 and CD14^+^ cells. **(C)** Percentages of HLA-G-expressing cells (top panels) and HLA-G MFI (bottom panels) on DC-10, CD11c^+^, and cDC1 cells. For **(B, C)**, each dot represents a single donor; lines connect the data of the same donor. Numbers indicate p-values of paired Wilcoxon’s test after Bonferroni’s correction. The correction was performed for all comparisons in **(B, C)**, separately for the type of data (either the percentage of HLA-G-expressing cells or the HLA-G MFI). Statistically significant p-values are indicated in bold.

**Figure 3:**
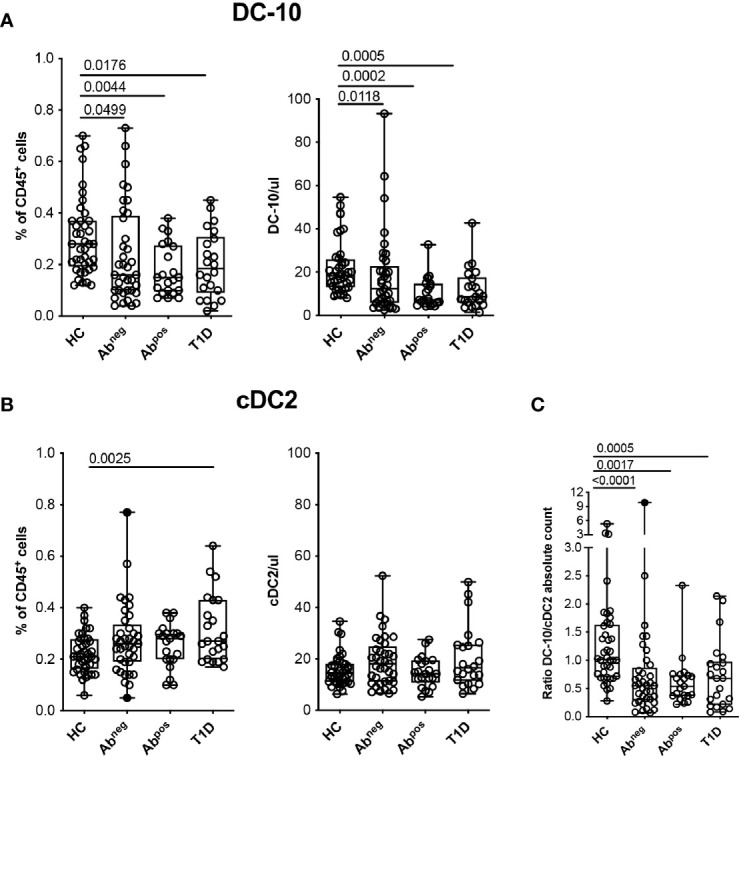
Tolerogenic DC-10 are reduced in type 1 diabetes (T1D) patients and in both Ab^neg^ and Ab^pos^ first-degree relative (FDR) subjects. DC-10 (CD11c^+^CD14^+^CD16^+^CD141^+^CD163^+^) and cDC2 (CD11c^+^CD1c^+^) were identified by multiparametric flow cytometry in the peripheral blood of pediatric healthy donors (HCs; n = 40), of age-matched first-degree relatives of T1D patients without (Ab^neg^, n = 37) or with (Ab^pos^, n = 21) autoantibodies, and pediatric T1D patients at onset (T1D, n = 22). The frequency and absolute cell count of **(A)** DC-10 and of **(B)** cDC2 in the indicated cohort of donors are shown. **(C)** Ratio between DC-10 and cDC2 as absolute cell count was calculated for each donor. The absolute cell counts of DC-10 and cDC2 were obtained by normalization of the CD45^+^ cells based on the full blood count laboratory test. Each dot represents a single donor, and black dots in the Ab^neg^ group indicate donors identified as outliers by the corresponding linear mixed-effects (LME) model used for the comparing groups; lines indicate medians, and whiskers are minimum and maximum levels. For each set of data, *post-hoc* analysis of the LME model with the R package phia was performed for testing the difference between all groups. Numbers indicate statistically significant Bonferroni’s adjusted p-values. The p-values of all comparisons are reported in **Supplementary Tables S2, S4**. In the LME analysis, data on the percentage of DC-10 and cDC2 cells were used in square root scale, while data on absolute cell counts (even the ratio DC-10/cDC2) were used in natural logarithmic scale in order to meet the assumptions of the model.

**Figure 4:**
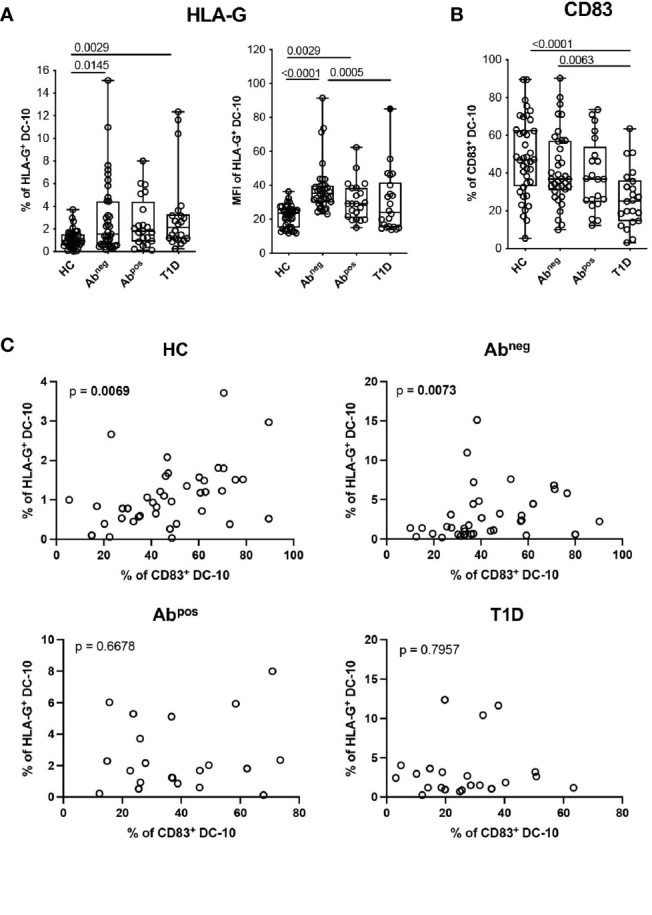
DC-10 from type 1 diabetes (T1D) patients and first-degree relative (FDR) subjects show an altered phenotype. DC-10 were identified as CD11c^+^CD14^+^CD16^+^CD141^+^CD163^+^, cells and the expression levels of the indicated markers were measured by multiparametric flow cytometry in the peripheral blood of pediatric healthy donors (HCs; n = 40), age-matched first-degree relatives of T1D patients without (Ab^neg^, n = 37) or with (Ab^pos^, n = 21) autoantibodies, and pediatric T1D patients (T1D, n = 22). **(A)** Percentages of HLA-G-expressing cells (left panel) and HLA-G MFI (right panel) on DC-10. **(B)** Percentages of CD83^+^ DC-10. Each dot represents a single donor, and the black dot in the T1D group indicates a donor identified as outlier by corresponding linear mixed-effects (LME) model used for the comparing groups; lines indicate medians, and whiskers are minimum and maximum levels. For each set of data, *post-hoc* analysis of the LME model with the R package phia was performed for testing the difference between groups. Numbers indicate statistically significant Bonferroni’s adjusted p-values. The p-values of all comparisons are reported in **Supplementary Table S2**. In the LME analysis, the % of HLA-G^+^ DC-10 and MFI of HLA-G^+^ DC-10 were used in natural logarithmic scale, while the % of CD83^+^ DC-10 were used in square root scale in order to meet the assumptions of the model. **(C)** Analysis of dependency between the expression of HLA-G and CD83 in DC-10 in the indicated cohorts of donors are shown. Data distribution and p-values are shown for each analysis. Statistically significant p-values are indicated in bold. In the analysis, the response variable (% of HLA-G^+^ DC-10) was used in square root scale for the HC and Ab^pos^ groups, and in natural logarithmic scale, for the Ab^neg^ and T1D groups in order to meet the assumptions of the model.

Statistical methods applied for each set of data in **Figures 1**, **2** are reported in the related figure legend. For **Figures 3**, **4A, B**, and **Supplementary Figures S3, S4C** and **S5**, comparisons among groups were performed with linear mixed-effects (LME) models to account for the presence of several subjects within the same family. In each analysis, to meet the assumption of normality of the residuals of the models, an appropriate transformation, indicated in the figure legend, was eventually applied to the response variable; and, when necessary, few outliers identified by the specific model were not included in that analysis. For the sake of completeness, these outliers have been included in the figures as black dots (**Figures 3B, C**, **Supplementary Figures S3A** and **S5**) and considered in the computation of the reported descriptive statistics. For testing difference between all pairs of groups, a post-hoc analysis was performed by using the R package phia and by applying Bonferroni’s correction to account for multiple comparisons. For (**Figure 4C**), the linear model was employed to evaluate the dependency between % of CD83^+^ DC-10 and % of HLA-G^+^ DC-10, within each group. The standard linear regression was estimated for the groups HC, Ab^pos^, and T1D, since all subjects were independent, while the LME model was used in case of Ab^neg^ in order to account for the presence of several subjects within the same family. In each analysis, in order to meet the assumption of normality of the residuals of the model, an appropriate transformation was applied to the response variable (% of HLA-G^+^ DC-10).

**Figure 5:**
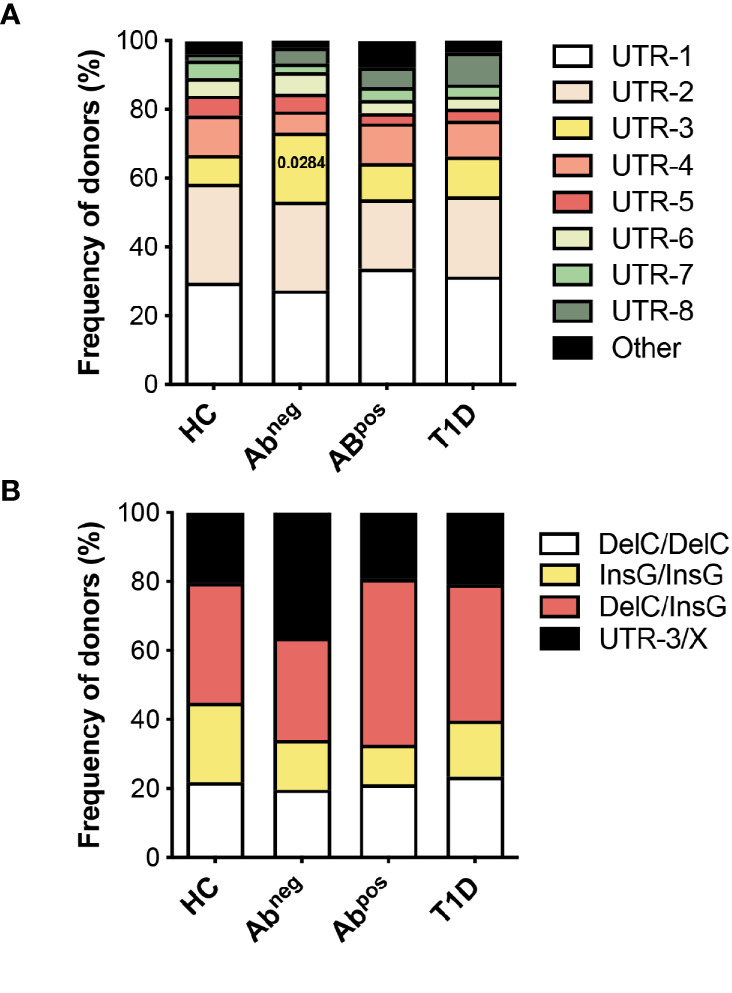
HLA-G UTR-3 is more represented in Ab^neg^ first-degree relatives (FDRs). **(A)** Allele relative frequencies based on the analysis of nine polymorphic sites present in the 3′UTR of HLA-G locus in healthy donors (HC; n = 78), first-degree relatives of type 1 diabetes (T1D) patients without (Ab^neg^, n = 97) or with (Ab^pos^, n = 52) autoantibodies, and T1D patients (T1D, n = 43) are shown. **(B)** Genotype relative frequencies for 14-bp Ins/Del and +3142 G/C polymorphisms present in the 3′UTR of HLA-G locus in the indicated cohorts of donors are shown. The logistic mixed-effects models were employed for comparing the relative frequency of the single haplotypes or genotypes of all groups *vs*. HCs, as described in the *Materials and Methods*. Numbers indicate statistically significant Bonferroni’s adjusted p-values. The p-values of all comparisons are reported in **Supplementary Table S5**.

For the genetic analyses of HLA-G 3′UTR and 5′URR (PROMO) regions reported in (**Figure 5**, **Supplementary Figure S6, Supplementary Tables S5** and **S7**), logistic mixed-effects models were employed for comparing the frequency of the single haplotypes or genotypes between each group HC, Ab^pos^, and T1D *versus* Ab^neg^. The models account for the presence of several subjects within the same family and, in case of the haplotypes, also for the presence of two alleles per subject. Comparisons have been performed only for haplotypes or genotypes with at least two groups with absolute frequency of at least 10. P-Values were adjusted by applying Bonferroni’s correction to account for multiple testing.

Multiple mixed-effects regression analyses were performed to identify potential predictive variables of T1D development, since these models can account for the presence of several individuals within the same family. The logistic mixed-effects model was employed for predicting the probability to be Ab^neg^ *versus* HCs. The cumulative link mixed model was used for predicting the probability to be: a subject at risk without (Ab^neg^) or with autoimmunity (Ab^pos^) or to be a person with T1D (considering the groups as ordered). For both analyses, the variables considered in the starting model were Age, % of DC-10, % of cDC2, % of HLA-G^+^ DC-10, % of CD83^+^ DC-10, and 3′UTR genotype (considering the comparisons of all genotypes with respect to DelC/DelC genotype). The final models were obtained with a backward procedure of variable selection.

In all the analyses, p-values less than 0.05 were considered significant. All p-values of the analyses were reported in the figures and/or (**Supplementary Tables S2, S5** and **S7**). Statistical analysis was performed using R 3.5.0 (http://www.R-project.org/).

## Results

### The Frequency of Circulating DC-10 Is Stable in Children and Adults

Using the newly identified set of markers CD14, CD16, CD141, and CD163 (14), and the gating strategy shown in (**Supplementary Figure S1A**), we evaluated the frequency of circulating DC-10 in a cohort of 55 healthy donors (HCs). The distribution of classical (c)DC1 (CD11c^+^CD14^−^BDCA-3^+^), cDC2 (CD11c^+^BDCA-1^+^), and plasmacytoid (p)DCs (CD11c^−^BDCA-2^+^) subsets (**Supplementary Figure S1B**) was analyzed in parallel. The frequency of DC-10 was constant with respect to the age among the donors analyzed and represented on average 0.31% ± 0.15% (range 0.07%–0.70%) of CD45^+^ cells (**Figure 1A**). Similarly, the frequency of cDC1 and cDC2 subsets was stable in young and adult HCs being on average 0.45% ± 0.20% (range 0.05%–1.19%) and 0.23% ± 0.09% (range 0.06%–0.49%) of CD45^+^ cells, respectively. In line with previous studies (18), the frequency of pDC decreased with age (r = −0.3061, p = 0.0231) and represented on average 0.17% ± 0.08% (range 0.05%–0.45%) of CD45^+^ cells (**Figure 1A**).

DC-10 are a subset of CD14^+^CD16^+^ cells (14); thus, we analyzed the frequency of circulating monocyte subsets according to the standard classification (19): classical (CD14^high^CD16^−^), CD14^+^CD16^+^, and non-classical (CD14^low^CD16^+^) monocytes. The proportion of non-classical monocytes decreased with age (r = −0.2872, p = 0.0335) with an average of 0.44% ± 0.21% (range 0.16%–0.95%) in CD45^+^ cells, while the frequencies of classical and CD14^+^CD16^+^ monocytes were constant in young and adult HCs, with an average of 2.55% ± 1.65% (range 0.31%–6.74%) and of 1.36% ± 1.02% (range 0.25%–4.72%) in CD45^+^ cells (**Figure 1B**).

Overall, in healthy conditions, the distribution of DC-10 in the peripheral blood is constant in young and adults.

### DC-10 Contain a Higher Proportion of HLA-G-Expressing Cells Than Do Total CD14^+^ Cells, CD11c^+^ Cells, and cDC1

*In vitro* differentiated DC-10 express HLA-G (7, 13); thus, we investigated whether this feature is shared by their *in vivo* counterpart. Circulating DC-10 from HCs expressed variable levels of HLA-G ranging from 0.03% to 24.37%, with the percentage of HLA-G-positive DC-10 correlating with HLA-G expression levels, indicated by the MFI (r = 0.4724, p = 0.0005) (**Figure 2A**).

CD14^+^ monocytes constitute the main subset of immune cells expressing HLA-G, although at low and variable levels [e.g., 0.12% ± 0.07% of HLA-G^+^CD14^+^ cells (20–24)]. In our cohort of HCs, circulating DC-10 expressed significantly higher HLA-G levels compared to those expressed by total CD14^+^ cells in terms of both percentage of HLA-G^+^ cells (2.19% ± 3.84% and 0.66% ± 0.93%, respectively, p < 0.0001) and levels of protein expression (MFI of HLA-G, 25.95% ± 10.82% and 26.23% ± 18.96%, respectively, p < 0.0001) (**Figure 2B** and **Supplementary Figure S2A**). The frequency of HLA-G^+^ DC-10 was also significantly higher compared with the percentage of HLA-G^+^CD11c^+^ (0.30% ± 0.37%, p < 0.0001) and of HLA-G^+^cDC1 cells (0.78% ± 1.17%, p = 0.0004) (**Figure 2C** and **Supplementary Figure S2B**). Moreover, HLA-G MFI on DC-10 was significantly higher compared with that on CD11c^+^ cells (32.55% ± 47.25%, p = 0.0015), but similar to that detected on cDC1 (31.84% ± 19.08%, p = 1.0000) (**Figure 2C** and **Supplementary Figure S2B**).

Altogether, these results indicate that DC-10 contain a higher percentage of HLA-G-expressing cells among the myeloid cell subsets analyzed.

### Tolerogenic DC-10 Are Reduced in Type 1 Diabetes patients at Onset and in Both Ab^neg^ and Ab^pos^ First-Degree Relative Subjects

Hypothesizing that DC-10 frequency could be altered in diseases characterized by breakage of tolerance, we investigated possible variations of DC-10 frequency in subjects with different stages of T1D. Glucose variability in long-standing T1D patients can affect myeloid and stem and progenitor cell compartments (25, 26); thus, we performed the analysis in pediatric new-onset T1D patients, in comparison with age-matched FDRs of T1D patients without or with autoantibodies (Ab^neg^ and Ab^pos^, respectively), and age-matched HCs (**Table 1**). Circulating DC-10 were significantly lower as frequency and as absolute cell count in T1D patients (0.20% ± 0.13%, p = 0.0176; and 12.20% ± 9.44 cells/ml, p = 0.0005, respectively), Ab^pos^ FDRs (0.18% ± 0.10%, p = 0.0044; and 10.53 ± 6.96 cells/ml, p = 0.0002, respectively), and Ab^neg^ FDRs (0.24% ± 0.19%, p = 0.0499; and 18.25 ± 18.87 cells/ml, p = 0.0118, respectively) compared with HCs (0.31% ± 0.15%, and 22.02 ± 12.22 cells/ml) (**Figure 3A** and **Supplementary Table S2**). The proportion, but not the total cell number, of classical cDC2 was significantly higher in T1D patients compared with HCs (0.32% ± 0.13% and 0.22% ± 0.07%, respectively; p = 0.0025) (**Figure 3B** and **Supplementary Table S2**). Notably, the DC-10/cDC2 absolute cell number ratio was significantly higher in HCs (1.31 ± 0.92) compared with Ab^neg^ FDRs (0.89 ± 1.59, p < 0.0001), Ab^pos^ FDRs (0.62 ± 0.45, p = 0.0017), and T1D patients (0.73 ± 0.60, p = 0.0005) (**Figure 3C** and **Supplementary Table S2**). No differences in the frequency of classical cDC1 and pDC were observed among the different cohorts (**Supplementary Figure S3A** and **Supplementary Table S2**). According to previous data (27, 28), the frequency of CD11c^+^HLA-DR^+^ myeloid DCs was reduced in T1D patients (36.60% ± 10.15%) compared with that present in HCs (47.06% ± 13.45%, p = 0.0107) and in Ab^neg^ FDRs (46.86% ± 14.00%, p = 0.0424) but was similar to that observed in Ab^pos^ FDRs (38.13% ± 14%) (**Supplementary Figure S3B** and **Supplementary Table S2**). Finally, in T1D patients, and in Ab^neg^ FDRs and Ab^pos^ FDR subjects, the frequency of the different monocyte subsets was comparable with that of HCs, except for classical monocytes being significantly higher in Ab^neg^ FDRs compared with HCs (3.57% ± 1.45% and 2.42% ± 1.65%, respectively; p = 0.0008) (**Supplementary Figure S3C** and **Supplementary Table S2**).

Overall, these findings indicate a specific reduction in the tolerogenic DC-10 compartment that is parallel to the increase of the pro-inflammatory cDC2 in T1D patients, Ab^neg^ FDRs, and Ab^pos^ FDRs.

### DC-10 From Type 1 Diabetes Patients and First-Degree Relatives Show an Altered Phenotype

We further characterized circulating DC-10 focusing on the expression of HLA-G and of ILT4, tolerogenic molecules associated with DC-10 regulatory activity (13, 14), and the activation marker CD83. A higher percentage of HLA-G^+^ DC-10 was identified in Ab^neg^ FDRs (2.96% ± 3.28%, p = 0.0145) and in T1D patients (3.24% ± 3.51%, p = 0.0029) and, as a tendency, in Ab^pos^ FDRs (2.56% ± 2.23%, p = 0.0593) compared with HCs (1.11% ± 0.78%) (**Figure 4A** and **Supplementary Table S2**). Notably, in Ab^neg^ FDRs, DC-10 expressed significantly higher levels of HLA-G (MFI of HLA-G 37.74% ± 14.16) than did HCs (MFI of HLA-G, 21.89 ± 6.41, p < 0.0001) and T1D patients (MFI of HLA-G 30.07 ± 17.90, p = 0.0005) (**Figure 4A** and **Supplementary Table S2**). Moreover, despite the high variability, Ab^neg^ FDRs and HCs showed on average the highest percentage of CD83**^+^** DC-10, which was significantly higher compared with that of T1D patients (42.17% ± 19.16% and 48.19% ± 20.59% *vs.* 26.61% ± 15.43%, p = 0.0063 and p < 0.0001, respectively) (**Figure 4B**, **Supplementary Figure S4A** and **Supplementary Table S2**). The percentage of CD83^+^CD11c^+^ and of CD83^+^ cDC1 cells was comparable among the groups analyzed (**Supplementary Figure S5** and **Supplementary Table S2**). Interestingly, the percentage of HLA-G^+^ DC-10 correlated with the percentage of CD83^+^ DC-10 in Ab^neg^ FDRs (p = 0.0073). The dependency between the percentage of HLA-G^+^ DC-10 and the percentage of CD83^+^ DC-10 was also detected in HCs (p = 0.0069) but not in Ab^pos^ FDRs or T1D patients (subjects with autoimmunity) (**Figure 4C**). No differences in the proportion of ILT4**^+^** DC-10 were detected among the different cohorts (**Supplementary Figures S4B, C** and **Supplementary Table S2**).

These findings indicate that in T1D patients and Ab^neg^ and Ab^pos^ FDRs, despite the lower proportion, DC-10 expressed HLA-G at higher levels compared with DC-10 from HCs. Moreover, in subjects with autoimmunity (e.g., T1D patients and Ab^pos^ FDRs), DC-10, but not other myeloid cell subsets, are less activated compared with those present in subjects without autoimmunity (e.g., Ab^neg^ FDRs and HCs).

### Ab^neg^ First-Degree Relative Subjects Differ From Age-Matched Healthy Controls

Our data indicate that in T1D patients at onset and in subjects at risk to develop the disease (FDRs), DC-10 number and phenotype are different from those in HCs. Moreover, in Ab^neg^ FDRs, despite that DC-10 are present at lower frequency compared with those in HCs, these cells expressed high levels of HLA-G (**Figure 4A**). An association between specific HLA-G genotypes/haplotypes and T1D has been previously demonstrated (10–12); thus, we analyzed nine specific variations of the 3′UTR HLA-G locus and inferred haplotypes and genotypes in the different cohorts of subjects (**Supplementary Tables S3** and **S4**).

To further define differences between Ab^neg^ FDRs and HCs, we performed multiple regression analysis considering the following variables: age of the persons, % of DC-10, % of cDC2, % of HLA-G^+^DC-10, % of CD83^+^DC-10, and 3′UTR HLA-G genotype (considering the comparisons InsG/InsG, DelC/InsG, and UTR-3/X genotypes with respect to the DelC/DelC genotype) (**Table 3**). Multiple regression analysis identified all variables investigated to significantly predict the probability of being Ab^neg^ FDRs, with the only exception of the DelC/InsG *vs.* DelC/DelC comparison (**Table 3**), indicating that overall Ab^neg^ FDR subjects differ from HCs with respect to these parameters. These findings confirm and further support previous evidence that Ab^neg^ FDRs cannot be considered healthy, as they already have signs of metabolic and immunological alterations (29–31).

**Table 3:** Multiple regression analysis to compare Ab^neg^ FDRs with healthy controls.

Variables	Coefficient	Standard error	p-Value
Intercept	39.379	18.919	**0.0374**
Age	−4.113	1.624	**0.0113**
% of DC-10	−131.960	48.966	**0.0070**
% of cDC2	86.396	42.981	**0.0444**
% of HLA-G^+^ DC-10	24.331	8.567	**0.0045**
% of CD83^+^ DC-10	−1.246	0.403	**0.0020**
3′UTR genotype			
InsG/InsG *vs.* DelC/DelC	59.319	22.684	**0.0089**
DelC/InsG *vs.* DelC/DelC	20.380	10.875	0.0609
UTR-3/X *vs.* DelC/DelC	23.947	11.107	**0.0311**

Final logistic mixed-effects model for predicting the type of group (individual at risk of develop T1D without autoimmunity, n = 37 Ab^neg^ FDRs vs. n = 40 healthy controls), by accounting for the presence of several subjects within the same family. The final model was obtained with a backward procedure of variable selection as described in the Material and Methods. Statistically significant p-values are indicated in bold.

FDRs, first-degree relatives; T1D, type 1 diabetes.

### The Frequency of CD83^+^ DC-10 Predicts the Probability to Be a Type 1 Diabetes Patient

To define whether the same set of variables that distinguish Ab^neg^ FDRs from HCs can predict the probability to be an individual at risk without (Ab^neg^) or with autoimmunity (Ab^pos^) or a T1D patient, considering the groups as ordered, we employed a cumulative link mixed model. The analysis showed that only the percentage of CD83^+^ DC-10 can be considered a significant risk factor to develop the disease (coefficient of the model = −0.0881, p = 0.0373). Indeed, a progressive decrease of CD83^+^ DC-10 percentage is associated with disease progression (e.g., from Ab^neg^ FDRs, to Ab^pos^ FDRs, to overt disease). The observation that none of the other variables, including the % of DC-10 or the % of HLA-G^+^ DC-10, were retained in the final cumulative link mixed model while they were retained in the previous final model predicting Ab^neg^ FDRs *vs.* HCs indicates that Ab^neg^ FDRs share several similarities with Ab^pos^ FDRs and with T1D patients, while they differ from HCs with respect to these variables.

### HLA-G UTR-3 Is More Represented in Ab^neg^ First-Degree Relative Subjects

The observations that specific 3′UTR HLA-G genotypes can predict the probability of being Ab^neg^ FDRs, and that Ab^neg^ FDRs are more similar to subjects with autoimmunity, prompted us to perform an additional HLA-G genetic analysis to explore if HLA-G genetic variations represent risk factors for developing T1D. To this aim, we enlarged the analysis by including adult subjects (**Table 2**), and by genotyping both the 3′UTR and promoter (PROMO) regions of HLA-G. The frequency of UTR-3 allele was significantly higher in Ab^neg^ FDRs (20.1%) compared with HCs (8.3%, p = 0.0283; **Figures 5A, B**, **Supplementary Table S5**) and comparable with the frequency assessed in Ab^pos^ FDRs (10.6%, p = 0.2603) and in T1D patients (11.6%, p = 0.5331). We also analyzed 28 different single-nucleotide polymorphisms in the PROMO region of HLA-G gene (**Supplementary Table S6**). We inferred almost a hundred different haplotypes, of which 16 was already described, six with a frequency >0.5%, and 78 with a frequency <0.5%, which clustered in four major PROMO families (data not shown). Among them, we observed a significantly higher frequency of the PROMO-0104 allele in Ab^neg^ FDRs compared with HCs (17.5% *vs.* 7.1%, p = 0.0390) (**Supplementary Figure S6** and **Supplementary Table S7**). These results, in conjunction with the high frequency of UTR-3 in Ab^neg^ FDRs, confirm previous data on the linkage disequilibrium between the 3′UTR-3 and the PROMO-0104 alleles (9). However, probably the smaller sample size of Ab^pos^ FDRs and T1D groups did not allow to define the protective role of PROMO-0104 alleles *versus* disease progression, although it was present with a lower percentage in these two groups with respect to Ab^neg^ FDRs (for Ab^pos^ FDRs: 6.7%, p = 0.0664; for T1D: 8.1%, p = 0.2073).

## Discussion

In this study, we report that naturally occurring DC-10 contain a higher frequency of HLA-G-expressing cells compared with CD14^+^, CD11c^+^, and cDC1 cells. Moreover, we show, for the first time, that the frequency and number of circulating DC-10 are reduced in pediatric T1D patients at onset and in individuals at risk of developing the disease, while the proportion of pro-inflammatory cDC2 cells increases in these groups. In addition, the frequency of CD83^+^DC-10 progressively decreases from Ab^neg^ FDRs to Ab^pos^ FDRs, to T1D patients, and a low proportion of CD83^+^DC-10 is associated with disease progression. Multiple regression analysis of variables related to DC-10 and HLA-G confirms that Ab^neg^ FDRs differ from healthy subjects and are more similar to T1D patients and to Ab^pos^ FDRs.

Thus far, the presence and the frequency of DC-10 have been described in the peripheral blood of adults (7, 13, 14, 32). The present study reveals that, similar to other myeloid DC subsets, the frequency of circulating DC-10 is stable throughout life and represents on average the 0.3% of total leucocytes, while the proportion of plasmacytoid DCs decreased in adults (18). The expression of HLA-G is a specific feature of *in vitro* differentiated DC-10 (7), and high HLA-G expression is a key determinant for their tolerogenic activity (13). Here, we show that, although the frequency of HLA-G^+^ DC-10 is variable among the donors analyzed, DC-10 contain a higher proportion of HLA-G-expressing cells compared with CD14^+^ monocytes, previously described to be the major myeloid population expressing/secreting HLA-G (21, 22, 33, 34), and to other DC subsets, such as CD11c^+^ and cDC1 cells.

Studies on the frequency of myeloid cells in T1D patients have been limited to pro-inflammatory cells, including DCs (27, 28, 35). We scrutinized for the first time the presence and the frequency of pro-inflammatory and anti-inflammatory APCs (e.g., DC-10) in pediatric Ab^neg^ FDRs and Ab^pos^ FDRs and in T1D patients at onset. We showed a reduced frequency and number of DC-10 in T1D patients and in subjects at risk to develop the disease compared with HCs. DC-10 reduction is paralleled with the increased frequency of pro-inflammatory cDC2, and the ratio DC-10/cDC2 progressively decreases from HCs to Ab^neg^ FDRs, Ab^pos^ FDRs, and T1D patients. The DC-10 contraction is specific since CD14^+^CD16^+^ monocytes, containing DC-10, are present at comparable frequency in the different cohorts analyzed independently from the stage of the disease.

The increased cDC2 frequency in T1D patients seems to be in contrast with two recent publications showing a reduction in the proportion of myeloid DCs in T1D patients compared with HCs (27, 28). However, the latter analysis was performed using different gating strategies and combinations of markers: myeloid DCs were defined as lin^−^HLA-DR^+^CD123^−^CD11c^+^ cells. Using the same strategy, we confirmed the reduced frequency of HLA-DR^+^CD11c^+^ cells in T1D patients compared with HCs. Notably, with our detailed analysis, which allows the segregation of myeloid HLA-DR^+^CD11c^+^ cells in CD1c/BDCA-1- and CD141/BDCA-3-expressing DCs, we showed the specific expansion of the former compartment, known to be the main producers of IL-12, tumor necrosis factor-α (TNF-α), IL-8, and IL-10, and inducers of cytotoxic T cells (36–38). This result supports the hypothesis that the enriched frequency of pro-inflammatory myeloid DCs is associated with the activation and differentiation of auto-reactive T cells sustaining T1D development. Moreover, our results suggest that the decrease of DC-10 and the DC-10/cDC2 ratio weighted toward the immunogenic compartment could be indicators of break of tolerance.

DC-10 from Ab^pos^ FDRs and T1D patients express higher levels of HLA-G, in terms of percentage both of positive cells and of protein expression, compared with HCs. Notably, the percentage of DC-10 expressing CD83 decreases significantly in T1D patients and, as a tendency, in Ab^pos^ FDRs when compared with both healthy subjects and Ab^neg^ FDRs. Correlation analysis between the percentage of HLA-G^+^ DC-10 and CD83^+^ DC-10 revealed a dependency between these two parameters in Ab^neg^ FDRs and in HCs but not in Ab^pos^ FDRs and in T1D patients. This result suggests a possible correlation between DC-10 phenotype and function that it is lost during disease progression. Multiple regression analyses supported this hypothesis, since it revealed that the percentage of CD83^+^ DC-10 not only discriminates between Ab^neg^ FDRs and HCs but also represents a risk factor for developing T1D. Indeed, a reduction of CD83^+^ DC-10 percentage is associated with disease progression. The downregulation of CD83 on DCs resulted in a lower stimulatory capacity (39); therefore, we can speculate that, despite the high expression of HLA-G, DC-10 in Ab^pos^ FDRs and in T1D patients are poorly activated and might have an impaired ability to activate and/or induce regulatory T cells. The decrease in CD83 expression is specific for DC-10, since other myeloid cells (e.g., CD11c^+^ and cDC1) expressed CD83 independently from the stage of the disease, suggesting that the latter pro-inflammatory cells are not impaired in their activation and possibly functions. Future dedicated functional studies using *ex vivo* isolated DC-10 from subjects at different stages of the disease are warranted to verify our hypothesis.

Single parameter and multiple regression analyses further support previous evidences that Ab^neg^ FDRs differ from HCs and cannot be considered healthy, as they already have signs of metabolic and immunological alteration such as altered neutrophil-specific and type I IFN signatures (29–31). Results from the cumulative link mixed analysis, demonstrating that Ab^neg^ FDRs are more similar to Ab^pos^ FDRs and to T1D patients, further sustain this conclusion. Indeed, several DC-10-related parameters, including the % of DC-10 and the % of HLA-G^+^ DC-10, can discriminate between Ab^neg^ FDRs and HCs, but they seem not to distinguish Ab^neg^ FDRs from Ab^pos^ FDRs and from T1D patients. Based on these findings, we can speculate that the proportion of DC-10 and of HLA-G^+^DC-10 can discern also Ab^pos^ FDR from HCs and T1D patients from HCs and could be considered risk factors to develop the disease. Further studies including larger cohorts of patients are warranted to confirm this hypothesis.

Genetic variants of the *HLA-G* locus have been associated with the risk to develop T1D (40), with contrasting results. Silva and colleagues reported an increased frequency of the 14 base pair deletion (14DEL) polymorphism in T1D patients compared with HCs (12). Conversely, a study performed considering HLA-G 3′UTR extended haplotypes described a lower frequency of the UTR-3 haplotype, containing the 14DEL polymorphism, in T1D patients compared with HCs (10). In the present study, we found a similar frequency of UTR-3 in T1D patients and HCs. However, the different genetic backgrounds derived from the geographical origin of our cohorts of patients compared with the study from Brazilian groups can influence the frequency of genetic variants and should be considered.

In conclusion, for the first time, we show an increase of the pro-inflammatory arm of the myeloid immunity (cDC2) and a reduction of the tolerogenic compartment (DC-10) in individuals at risk of developing T1D and in T1D patients at onset. Interestingly, the monitoring of the myeloid compartment, and, in particular, of CD83^+^ DC-10, might be used to follow changes in the immunological status from the absence of signs of autoimmunity to the overt disease. Moreover, the high frequency of HLA-G UTR-3, previously shown to be associated with HLA-G expression levels, in individuals at risk to develop T1D without signs of autoimmunity (Ab^neg^ FDRs) could represent a protective factor toward the disease development. These results pave the way to additional longitudinal studies, based on longer follow-up and including larger cohorts of subjects, to investigate the functional role of DC-10 in controlling/preventing T1D development and to further define if HLA-G genetics, in addition to the HLA-DR/DQ screening, can be used as an additional risk/protection factor for disease development.

## Data Availability Statement

The original contributions presented in the study are included in the article/**Supplementary Material**. Further inquiries can be directed to the corresponding author.

## Ethics Statement

The studies involving human participants were reviewed and approved by Ospedale San Raffaele ethics committee: approval IRB#DRI-003 and IRB#NHPROT32803-TN01. Written informed consent to participate in this study was provided by the participants’ legal guardian/next of kin.

## Author Contributions

GA designed and performed the experiments, analyzed and interpreted the data, and wrote the manuscript. AM contributed to the analysis of the data. RC performed some of the experiments. AS performed sample processing and biobanking. PMVR performed the statistical analysis and CDS supervised them. RB, MP, and EB provided the samples. MB conceived the scientific idea and helped in the data interpretation. SG conceived the scientific idea, supervised the experimental design and data interpretation, and wrote the manuscript. SG is the guarantor of this work and, as such, had full access to all the data in the study and takes responsibility for the integrity of the data and the accuracy of the data analysis. All authors contributed to the article and approved the submitted version.

## Funding

This work was supported by research funding from the Italian Telethon Foundation (TGT16-17G01), by the JDRF Innovation grant 1-PNF-2015-110-S-B to SG, and by the JDRF Strategic Research Agreement (SRA)—JDRF International TrialNet Clinical Center in Italy (JDRF Grant Key: 3-SRA 2015-29-Q-R) to EB.

## Conflict of Interest

The authors declare that the research was conducted in the absence of any commercial or financial relationships that could be construed as a potential conflict of interest.

## Publisher’s Note

All claims expressed in this article are solely those of the authors and do not necessarily represent those of their affiliated organizations, or those of the publisher, the editors and the reviewers. Any product that may be evaluated in this article, or claim that may be made by its manufacturer, is not guaranteed or endorsed by the publisher.
