# Supplementary material for: Altered Frequency and Phenotype of HLA-G-Expressing DC-10 in Type 1 Diabetes Patients at Onset and in Subjects at Risk to Develop the Disease
Source: Front Immunol. 2021 Oct 1;12:750162. doi: 10.3389/fimmu.2021.750162 (PMC8517474; doi:10.3389/fimmu.2021.750162)
Supplement: Supplementary file 1 [file DataSheet_1.docx]

Supplementary Material

# Supplementary Methods

## DNA isolation and sequencing

Genomic DNA was isolated from EDTA peripheral blood using a commercial kit (QIAamp, QIAGEN, Germany) according to the manufacturer’ s instructions. DNA samples were stored at -20 °C.

## Ampliﬁcation and sequencing of HLA-G 3’ Un-translated region (UTR)

100 ng of genomic DNA were ampliﬁed in a 25 μl reaction containing 1x polymerase chain reaction (PCR) buffer, 0.2 mM dNTP mix, 1.5 mM MgCl2, 2 U Taq Polymerase (Applied biosystems, CA, USA), and 1μM of each primer (For: 5’TCACCCCTCACTGTGACTGA3’; Rev:5’ TTCTCATGTCTTCCATTTATTTTGTC3’, (Metabion, Germany). The initial denaturation step was carried out at 95 °C for 15 min, followed by 30 cycles at 93 °C for 60 s, annealing at 58 °C for 60 s, extension at 72 °C for 60 s, and by a ﬁnal extension step at 72 °C for 10 min. The ampliﬁcation product was evaluated using a 2% agarose gel, and puriﬁed using a commercial kit (QIAquick PCR purification kit, QIAGEN, Germany) according to the manufacturer’ s instructions. The Rev primer (5’TTCTCATGTCTTCCATTTATTTTGTC3’) was used to perform direct Sanger sequencing on both strands of puriﬁed ampliﬁcation products (Eurofins GATC, Germany). All the polymorphic sites included in the analysis are listed in **Supplementary Table S3**.

## Ampliﬁcation and sequencing of HLA-G promoter region (PROMO)

The amplification of the HLA-G promoter region was performed by two different PCR. The following primers were used: PF2 (5'CTGAACACTTACAACTGTGAGG3') and PR2 (5' CCGACACAGGTTAGGAGAAGG3'), PF4 (5'GATACGCACACTAGTTAC3') and PR4 (5'CCAGGACATCTCCTCAAG3') (Metabion, Germany). For each PCR, 100 ng of genomic DNA were ampliﬁed in a 25 μl reaction containing 1x polymerase chain reaction (PCR) buffer, 0.2 mM dNTP mix, 1.5 mM MgCl2, 2 U Taq Polymerase (Applied biosystems, CA, USA), and 1μM of each forward and reverse primer. The initial denaturation step was carried out at 95 °C for 2 min, followed by 35 cycles at 94 °C for 30 s, annealing at 60 °C (56 °C for the PF4/PR4) for 30 s, extension at 72 °C for 30 s, and by a ﬁnal extension step at 72 °C for 1 min. The ampliﬁcation product was evaluated using a 2% agarose gel, and puriﬁed using a commercial kit (QIAquick PCR purification kit, QIAGEN, Germany) according to the manufacturer’ s instructions. The PF2 and PF4 primers were used to perform direct Sanger sequencing on both strands of puriﬁed ampliﬁcation products (Eurofins GATC, Germany). All the polymorphic sites included in the analysis are listed in **Supplementary Table S6.**

## Haplotype assignment of HLA-G 3’ UTR and PROMO region

All the sequence products obtained were aligned and variation sites were individually annotated in house using the CodonCode Aligner software (Centerville, MA). The PHASE ([http://stephenslab.uchicago.edu/software.html](http://stepenslab.uchicago.edu/software.html)) method (1), implemented by the PHASE v2.1 package (Mac OS version) was used to assign the most probable haplotype constitution of each sample. Haplotypes and genotypes were named according to previous reports (2-4).

# References

1. Stephens M, Smith NJ, Donnelly P. A new statistical method for haplotype reconstruction from population data. Am J Hum Genet. 2001;68(4):978-89.

2. Amodio G, Mugione A, Sanchez AM, Vigano P, Candiani M, Somigliana E, et al. HLA-G expressing DC-10 and CD4(+) T cells accumulate in human decidua during pregnancy. Hum Immunol. 2013;74(4):406-11.

3. Castelli EC, Ramalho J, Porto IO, Lima TH, Felicio LP, Sabbagh A, et al. Insights into HLA-G Genetics Provided by Worldwide Haplotype Diversity. Front Immunol. 2014;5:476.

4. Martelli-Palomino G, Pancotto JA, Muniz YC, Mendes-Junior CT, Castelli EC, Massaro JD, et al. Polymorphic sites at the 3' untranslated region of the HLA-G gene are associated with differential hla-g soluble levels in the Brazilian and French population. PLoS One. 2013;8(10):e71742.

# Supplementary Tables

## Supplementary Table 1. List of antibodies used for cell identification by flow cytometry

| **Antigen** | **Clone** | **Fluorophore** | **Manufacturer** |
| --- | --- | --- | --- |
| CD11c | B-ly6 | FITC/PE-Cy7 | BD Bioscience, CA, USA |
| CD14 | MφP9 | APC-H7 | BD Bioscience, CA, USA |
| CD16 | 3G8 | BV510 | BD Bioscience, CA, USA |
| CD83 | HB15e | PE-Cy7 | BD Bioscience, CA, USA |
| CD141 (BDCA-3) | 1A4 | BV421 | BD Bioscience, CA, USA |
| CD163 | GHI/61 | PerCp 5.5 | BD Bioscience, CA, USA |
| ILT4 | 287219 | APC | BD Bioscience, CA, USA |
| HLA-G | 87G | PE | Exbio, Czech Republic |
| CD303 (BDCA-2) | AC144 | FITC | Miltenyi Biotech, Germany |
| HLA-DR | G46-6 | APC-H7 | BD Bioscience, CA, USA |
| CD1c (BDCA-1) | AD5-8E7 | PE | Miltenyi Biotech, Germany |

## Supplementary Table 2. P-values of post-hoc comparisons of Figure 3, Figure 4, Supplementary Figure 3, Supplementary Figure 4, and Supplementary Figure 5.

| **variable** | **P-value**  **HC vs Ab^Neg^** | **P-value**  **HC vs Ab^Pos^** | **P-value**  **HC vs T1D** | **P-value**  **Ab^Neg^ vs Ab^Pos^** | **P-value**  **Ab^Neg^ vs T1D** | **P-value**  **Ab^Pos^ vs T1D** |
| --- | --- | --- | --- | --- | --- | --- |
| **Figure 3A** | | | | | | |
| % of DC-10 | **0.0499** | **0.0044** | **0.0176** | 1.0000 | 1.0000 | 1.0000 |
| DC-10/ul | **0.0118** | **0.0002** | **0.0005** | 1.0000 | 1.0000 | 1.0000 |
| **Figure 3B** | | | | | | |
| % of cDC2 | 0.2595 | 1.0000 | **0.0025** | 1.0000 | 0.5093 | 0.2643 |
| cDC2/ul | 0.0596 | 1.0000 | 1.0000 | 0.0695 | 0.4570 | 1.0000 |
| **Figure 3C** | | | | | | |
| Ratio DC-10/cDC2 absolute count | **<0.0001** | **0.0017** | **0.0005** | 1.0000 | 1.0000 | 1.0000 |
| **Figure 4A** | | | | | | |
| % of HLA-G^+^ DC-10 | **0.0145** | 0.0593 | **0.0029** | 1.0000 | 1.0000 | 1.0000 |
| MFI of HLA-G^+^ DC-10 | **<0.0001** | **0.0029** | 0.5057 | 0.1741 | **0.0005** | 0.7379 |
| **Figure 4B** | | | | | | |
| % of CD83^+^ DC-10 | 1.0000 | 0.5755 | **<0.0001** | 1.0000 | **0.0063** | 0.0967 |
| **Figure S3A** | | | | | | |
| % of cDC1 | 1.0000 | 1.0000 | 1.0000 | 1.0000 | 0.8485 | 1.0000 |
| % of pDC | 1.0000 | 1.0000 | 1.0000 | 1.0000 | 1.0000 | 0.6873 |
| **Figure S3B** | | | | | | |
| % of CD11c^+^HLA-DR^+^ | 1.0000 | 0.0517 | **0.0107** | 0.1533 | **0.0424** | 1.0000 |
| **Figure S3C** |  |  |  |  |  |  |
| % of CD14^+^CD16^+^ cells | 1.0000 | 1.0000 | 0.6945 | 1.0000 | 1.0000 | 1.0000 |
| % of Non-classical | 0.2085 | 0.3121 | 0.0583 | 1.0000 | 1.0000 | 1.0000 |
| % of classical | **0.0008** | 1.0000 | 1.0000 | 0.0712 | 0.1094 | 1.0000 |
| **Figure S4C** | | | | | | |
| % of ILT4^+^ DC-10 | 1.0000 | 1.0000 | 1.0000 | 1.0000 | 1.0000 | 1.0000 |
| **Figure S5** | | | | | | |
| % of CD83^+^CD11c^+^ | 0.1207 | 1.000 | 1.000 | 1.000 | 0.4864 | 1.000 |
| % of CD83^+^ cDC1 | 1.000 | 0.1574 | 0.0637 | 1.000 | 1.000 | 1.000 |

For each variable, post-hoc analysis of the linear mixed-effects (LME) model for testing the difference between groups was performed (as described in the material and methods and in the corresponding figure). Adjusted p-values after Bonferroni’s correction are indicated. Statistically significant p-values are indicated in bold.

## Supplementary Table 3. List of polymorphic sites in the HLA-G 3’ Untranslated region (UTR) analyzed.

| **Genomic position**  **hg19 (Chr6)** | **SNPid** | **HLA-G position** | **Allele 1** | **Allele 2** |
| --- | --- | --- | --- | --- |
| 29798581 | rs371194629 | 2960 | G | GATTTGTTCATGCCT |
| 29798608 |  | 3001 | C | T |
| 29798610 | rs1707 | 3003 | C | T |
| 29798617 | rs1710 | 3010 | G | C |
| 29798634 | rs17179101 | 3027 | C | A |
| 29798642 | rs17179108 | 3035 | C | T |
| 29798749 | rs1063320 | 3142 | C | G |
| 29798794 | rs9380142 | 3187 | A | G |
| 29798803 | rs1610696 | 3196 | C | G |

## Supplementary Table 4. Estimated haplotype and genotype frequencies at HLA-G 3’ Un-translated region (UTR) polymorphic sites in the pediatric cohort used for the comparisons among groups

| **Haplotypes** | **HC**  **(n= 40)** | **Ab^neg^**  **(n = 37)** | **Ab^pos^**  **(n = 21)** | **T1D**  **(n = 22)** |
| --- | --- | --- | --- | --- |
| UTR-1 | 30 (37.5) | 20 (27.0) | 16 (38.1) | 13 (29.5) |
| UTR-2 | 19 (23.8) | 25 (33.8) | 6 (14.3) | 7 (15.9) |
| UTR-3 | 7 (8.8) | 13 (17.6) | 5 (11.9) | 6 (13.6) |
| UTR-4 | 7 (8.8) | 5 (6.8) | 5 (11.9) | 5 (11.4) |
| UTR-5 | 5 (6.2) | 1 (1.3) | 2 (4.8) | 2 (4.5) |
| UTR-6 | 3 (3.7) | 4 (5.4) | 3 (7.1) | 2 (4.5) |
| UTR-7 | 4 (5.0) | 0 (0) | 0 (0) | 2 (4.5) |
| UTR-8 | 1 (1.2) | 4 (5.4) | 4 (9.5) | 5 (11.4) |
| Others | 4 (5.0) | 2 (2.7) | 1 (2.4) | 2 (4.5) |
| **Genotypes** |  |  |  |  |
| DelC/DelC | 9 (22.5) | 7 (18.9) | 5 (23.8) | 5 (22.7) |
| InsG/InsG | 6 (15.0) | 6 (16.2) | 0 (0) | 3 (13.6) |
| DelC/InsG | 16 (40.0) | 12 (32.4) | 11 (52.4) | 8 (36.4) |
| UTR-3/X | 9 (22.5) | 12 (32.4) | 5 (23.8) | 6 (27.3) |

Allele and genotype absolute and relative frequencies (%) in the indicated groups of individuals are reported. “Others” are alleles previously described but present at low frequency.

## Supplementary Table 5. Estimated haplotype and genotype frequencies at HLA-G 3’ Untranslated region (UTR) polymorphic sites

| **Haplotypes** | **HC**  **(n= 78)** | **Ab^neg^**  **(n = 97)** | **Ab^pos^**  **(n = 52)** | **T1D**  **(n = 43)** | **P-value**  **HC vs Ab^neg^** | **P-value**  **Ab^pos^ vs Ab^neg^** | **P-value**  **T1D vs Ab^neg^** |
| --- | --- | --- | --- | --- | --- | --- | --- |
| UTR-1 | 46 (29.5) | 53 (27.3) | 35 (33.6) | 27 (31.4) | 1.0000/0.664* | 1.0000/0.2702* | 1.0000/0.4928* |
| UTR-2 | 45 (28.8) | 50 (25.8) | 21 (20.2) | 20 (23.2) | 1.0000/0.5424* | 1.0000/0.3648* | 1.0000/0.6350* |
| UTR-3 | 13 (8.3) | 39 (20.1) | 11 (10.6) | 10 (11.6) | **0.0283**/**0.0071*** | 0.2603/0.0651* | 0.5331/0.1333* |
| UTR-4 | 18 (11.5) | 12 (6.2) | 12 (11.5) | 9 (10.5) | 0.4161/0.1040* | 0.6745/0.1686* | 1.0000/0.2510* |
| UTR-5 | 9 (5.8) | 10 (5.1) | 3 (2.9) | 3 (3.5) |  |  |  |
| UTR-6 | 8 (5.1) | 12 (6.2) | 4 (3.8) | 3 (3.5) |  |  |  |
| UTR-7 | 8 (5.1) | 5 (2.6) | 4 (3.8) | 3 (3.5) |  |  |  |
| UTR-8 | 3 (1.9) | 9 (4.6) | 6 (5.8) | 8 (9.3) |  |  |  |
| Others | 6 (3.8) | 4 (2.1) | 8 (7.7) | 3 (3.5) |  |  |  |
| DelC/DelC | 17 (21.8) | 19 (19.6) | 11 (21.2) | 10 (23.3) | 1.0000/0.9221* | 1.0000/0.6757* | 1.0000/0.8909* |
| InsG/InsG | 18 (23.1) | 14 (14.4) | 6 (11.5) | 7 (16.3) | 1.0000/0.8396* | 1.0000/0.8211* | 1.0000/0.5140* |
| DelC/InsG | 27 (34.6) | 28 (29.6) | 25 (48.1) | 17 (39.5) | 1.0000/0.4240* | 0.1119/**0.0280*** | 0.8775/0.2194* |
| UTR-3/X | 16 (20.5) | 36 (37.1) | 10 (19.2) | 9 (20.9) | 0.1660/**0.0415*** | 0.2033/0.0508* | 0.3768/0.0942* |

Allele and genotype absolute and relative frequencies (%) in the indicated groups of individuals are reported. “Others” are alleles previously described but present at low frequency. The logistic mixed-effects models were employed for comparing the frequency of the haplotypes or genotypes between each group, healthy controls (HC), Ab^pos^, and T1D patients vs Ab^neg^. The models account for the presence of several subjects within the same family and, in case of the haplotypes, also for the presence of two alleles per subject. Comparisons have been performed only for haplotypes with at least 2 groups with absolute frequency of at least 10. P-values were adjusted by applying Bonferroni’s correction in order to account for multiple testing. Adjusted p-values after Bonferroni’s correction are reported and *indicates the p-values before the Bonferroni’s correction. Statistically significant p-values are in bold.

## Supplementary Table 6. List of polymorphic sites in the HLA-G Promoter region (PROMO) analyzed.

| **Genomic position**  **hg19 (Chr6)** | **SNPid** | **HLA-G position** | **Allele 1** | **Allele 2** | **Allele 3** |
| --- | --- | --- | --- | --- | --- |
| 29794317 | rs1736936 | -1305 | G | A |  |
| 29794443 | rs1736935 | -1179 | A | G |  |
| 29794467 | rs3823321 | -1155 | G | A |  |
| 29794482 | rs1736934 | -1140 | A | T |  |
| 29794484 | rs17875389 | -1138 | A | G |  |
| 29794501 | rs3115630 | -1121 | T | C |  |
| 29794524 | rs146374870 | -1098 | G | A |  |
| 29794658 | rs1632947 | -964 | G | A |  |
| 29794700 | rs370338057 | -922 | C | A |  |
| 29794812 | rs182801644 | -810 | C | T |  |
| 29794860 | rs1632946 | -762 | C | T |  |
| 29794897 | rs1233334 | -725 | G | C | T |
| 29794906 | rs2249863 | -716 | T | G |  |
| 29794933 | rs2735022 | -689 | A | G |  |
| 29794956 | rs35674592 | -666 | G | T |  |
| 29794976 | rs17875391 | -646 | A | G |  |
| 29794989 | rs1632944 | -633 | G | A |  |
| 29795076 | rs201221694 | -546/-540 | A | AG |  |
| 29795113 | rs17875393 | -509 | C | G |  |
| 29795136 | rs1736933 | -486 | A | C |  |
| 29795139 | rs149890776 | -483 | A | G |  |
| 29795145 | rs1736932 | -477 | C | G |  |
| 29795179 | rs17875394 | -443 | G | A |  |
| 29795222 | rs17875395 | -400 | G | A |  |
| 29795231 | rs17875396 | -391 | G | A |  |
| 29795253 | rs1632943 | -369 | C | A |  |
| 29795267 | rs191630481 | -355 | G | A |  |
| 29795421 | rs1233333 | -201 | G | A |  |

## Supplementary Table 7. Estimated haplotype and genotype frequencies at Promoter region (PROMO) polymorphic sites

| **Haplotypes** | **HC**  **(n = 78)** | **Ab^neg^**  **(n = 97)** | **Ab^pos^**  **(n = 52)** | **T1D**  **(n = 43)** | **P-value**  **HC vs Ab^neg^** | **P-value**  **Ab^pos^ vs Ab^neg^** | **P-value**  **T1D vs Ab^neg^** |
| --- | --- | --- | --- | --- | --- | --- | --- |
| 010101 | 53 (34.0) | 65 (33.5) | 42 (40.4) | 39 (45.4) | 1.0000/0.9331* | 0.7544/0.2515* | 0.2085/0.0695* |
| 010102 | 49 (31.4) | 63 (32.5) | 36 (34.6) | 29 (33.7) | 1.0000/0.8342* | 1.0000/0.7163* | 1.0000/0.8792* |
| 0103 | 3 (1.9) | 5 (2.6) | 2 (1.9) | 3 (3.5) |  |  |  |
| 0104 | 11 (7.1) | 34 (17.5) | 7 (6.7) | 7 (8.1) | **0.0391**/**0.0130*** | 0.0664/**0.0221*** | 0.2073/0.0691* |
| Undefined | 40 (25.6) | 27 (13.9) | 17 (16.4) | 8 (9.3) |  |  |  |

Allele absolute and relative frequencies (%) in the indicated groups of individuals are reported. “Undefined” are alleles present at low frequency e not previously described. The logistic mixed-effects models were employed for comparing the relative frequency of the single haplotypes between each group healthy controls (HC), Ab^pos^, and T1D patients vs Ab^neg^. The models account for the presence of several subjects within the same family and for the presence of two alleles per subject. Comparisons have been performed only for haplotypes with at least 2 groups with absolute frequency of at least 10. P-values were adjusted by applying Bonferroni’s correction in order to account for multiple testing. Adjusted p-values after Bonferroni’s correction are reported and *indicates the p-values before the Bonferroni’s correction. Statistically significant p-values are in bold.

# Supplementary Figures

## Supplementary Figure 1

**Supplementary Figure 1. Gating strategy to identify DC subsets and CD14^+^ monocytes in peripheral blood of healthy donors by flow cytometry.**

DC subsets were identified in the peripheral blood according to FSCarea/SSCarea physical parameters. (**A)** DC-10 were defined according to CD11c, CD14, CD16, CD141 and CD163 co-expression. (**B)** cDC1 were identified according to the lack of CD14 expression and CD11c and CD141 co-expression; cDC2 were identified according to CD11c and CD1c co-expression; pDC were identified according to the lack of CD11c expression and CD303 expression. The expression of HLA-DR was evaluated for cDC1, cDC2, and pDC in parallel. Percentages indicate the frequency of the different populations in the physical parameters. Dot plots from one representative donor are shown.

## Supplementary Figure 2

**Supplementary Figure 2. DC-10 contain a higher proportion of HLA-G-expressing cells compared to total CD14^+^ cells, CD11c^+^ cells, and cDC1.** Expression levels of HLA-G were evaluated by multiparametric flow cytometry in DC-10 (CD11c**^+^**CD14**^+^**CD16**^+^**CD141**^+^**CD163**^+^**) (**A**), CD14**^+^** monocytes (**A)**, CD11c**^+^** cells (**B**), and cDC1 (CD11c**^+^**CD14^-^CD141**^+^**) cells (**B)**. The percentages of HLA-G positive cells in the indicated cell populations from one representative donor are shown.

## Supplementary Figure 3

**Supplementary Figure 3. DC and monocytic subsets are present at comparable frequency in T1D patients and in FDRs subjects.** cDC1 (CD11c^+^CD14^-^CD141^+^), pDC (CD11c^-^CD303^+^), CD11^+^HLA-DR^+^ cells, and CD14^+^CD16^+^, non-classical (CD14^low^CD16^+^), and classical (CD14^high^CD16^-^) monocytes were identified by multiparametric flow cytometry in the peripheral blood of pediatric healthy donors (HC, n=40), of age matched T1D patients first degree relatives without (Ab^neg^, n=37) or with (Ab^pos^, n=21) autoantibodies, and pediatric T1D patients (T1D, n=22). The percentage of (**A**) cDC1 and pDC, (**B**) CD11^+^HLA-DR^+^ cells, and of (**C**) CD14^+^CD16^+^, non-classical, and classical monocytes in the indicated cohort of donors are shown. Each dot represents a single donor and the black dot in the pDC Ab^neg^ group indicates a donor identified as outlier by the corresponding linear mixed-effects (LME) model used for the comparing groups. Lines indicate medians, and whiskers are minimum and maximum levels. For each set of data, post-hoc analysis of the LME model with the R package phia was performed for testing the difference between groups. Numbers indicate statistically significant Bonferroni’s adjusted p-values. The p-values of all comparisons are reported in **Supplementary Table S2.** In the LME analysis, CD14^+^CD16^+^, non-classical, and classical monocytes data were used in natural logarithmic scale, while the percentage of cDC1 and of pDC cells were used in square root scale in order to meet the assumptions of the model.

## Supplementary Figure 4

**Supplementary Figure 4. DC-10 from T1D patients show an altered phenotype.** Expression levels of CD83 and of ILT4 were evaluated by multiparametric flow cytometry in DC-10 (CD11c^+^CD14^+^CD16^+^CD141^+^CD163^+^) in the peripheral blood of pediatric healthy donors (HC, n=40), age matched first degree relatives of T1D patients without (Ab^neg^, n=37) or with (Ab^pos^, n=21) autoantibodies, and pediatric T1D patients (T1D, n=22). Percentages of DC-10 positive for (**A**) CD83 and (**B**) ILT4 in one representative T1D patient and healthy control are shown. Filled line indicate the relative fluorescence minus one (FMO) staining. (**C**) Percentages of ILT4^+^ DC-10 in the indicated cohorts of donors are shown. Each dot represents a single donor; lines indicate medians, and whiskers are minimum and maximum levels. For each set of data, post-hoc analysis of the LME model with the R package phia was performed for testing the difference between groups. The p-values of all comparisons are reported in **Supplementary Table S2**. In the LME analysis, the % of ILT4^+^ DC-10 was used in square root scale in order to meet the assumptions of the model.

## Supplementary Figure 5

**Supplementary Figure 5. CD83 expression in the myeloid cell compartment.** Expression levels of CD83 were measured by multiparametric flow cytometry in the peripheral blood of pediatric healthy donors (HC, n=40), age matched first degree relatives of T1D patients without (Ab^neg^, n=37) or with (Ab^pos^, n=21) autoantibodies, and pediatric T1D patients (T1D, n=22). Percentages of CD83^+^ cells in CD11c^+^ cells (left panel) and in cDC1 (CD11c^+^CD14^-^CD141^+^, right panel) in the indicated cohort of donors are shown. Each dot represents a single donor, and the black dots in the Ab^neg^ and in T1D groups indicate donors identified as outlier by corresponding linear mixed-effects (LME) model used for the comparing groups; lines indicate medians, and whiskers are minimum and maximum levels. For each set of data, post-hoc analysis of the LME model with the R package phia was performed for testing the difference between groups. Numbers indicate statistically significant Bonferroni’s adjusted p-values. The p-values of all comparisons are reported in **Supplementary Table S2**. In the LME analysis, the % of CD83^+^CD11c^+^ were used in square root scale and the % of CD83^+^cDC1 were used in natural logarithmic scale, in order to meet the assumptions of the model.

## Supplementary Figure 6

**Supplementary Figure 6. PROMO-0104 haplotype family is more represented in Ab^neg^ FDRs.** Allele relative frequencies based on the analysis of 28 polymorphic sites present in the 5’ PROMO region of HLA-G locus in healthy donors (HC, n=78), first degree relatives of T1D patients without (Ab^neg^, n=97) or with (Ab^pos^, n=52) autoantibodies, and T1D patients (T1D, n=43). The logistic mixed-effects model was employed for comparing the relative frequency of the single haplotypes of all group vs Ab^neg^, as described in materials and methods. Numbers indicate statistically significant Bonferroni’s adjusted p-values. The p-values of all comparisons are reported in **Supplementary Table S7**.
